# “He needs to die in the hospital”: A caregiver’s distress call

**DOI:** 10.1017/S1478951525101351

**Published:** 2025-12-12

**Authors:** Monica Agosta, Juleen Min

**Affiliations:** Department of Palliative, Rehabilitation and Integrative Medicine, The University of Texas MD Anderson Cancer Center, Houston, TX, USA

**Keywords:** Case report, palliative care, end of life care, caregiver burden, hospital death

## Abstract

The setting in which patients face the end of life (EOL) is shaped by complex, individualized needs beyond physical care. Mr. X, a man in his mid-50s with glioblastoma, endured treatment complications and progressive decline. Supportive care was engaged to manage symptoms and support his wife, Mrs. X, who was simultaneously the family’s financial provider and caregiver for both her husband and their autistic adult son. As his condition worsened, she resisted hospice and code status changes, citing emotional, logistical, and financial barriers, while fiercely advocating for his wish to die in the hospital. Counseling and empathic validation helped her share her burdens and reframe her advocacy as rooted in love and grief. She ultimately consented to comfort-focused care, and Mr. X died peacefully in the hospital after a 31-day stay. This case underscores the need to integrate psychosocial factors into individualized EOL planning and to strengthen person-centered care.

## Introduction

When a patient approaches the end of life, multiple factors are considered when determining the best place of death (Vidal et al. 2022). Although most patients and caregivers prefer a home death, some believe the inpatient hospital setting is the best place for patient care (Jfv et al. 2022; Vidal et al. 2022). With advanced cancer patients, physical, psychological, financial, and other social concerns impact their preferred place of death (De Roo et al. 2014; Vidal et al. 2022). Additionally, the context in which a home death is presented to them can impact the decision of many patients and families (Valentino et al. 2023). When asked about preferences for a home death in the context of priorities, dying at home fell well below numerous other factors for patients with advanced cancer (Delgado-Guay et al. 2016). There is a dilemma when the patient no longer qualifies for reimbursement criteria for acute medical care, but the patient’s need exceeds what caregivers can provide, especially in the end-of-life (EOL) setting (Agar et al. 2008).

Although inpatient acute palliative care and hospice units play roles in symptom management that cannot be accommodated at home, they are not considered a permanent setting for patients to stay until death (Vidal et al. 2022; Wachterman et al. 2022). As supportive and palliative care specialists, we face caregiver distress related to the disposition involving multiple factors.

Herein, we present a case that underscores the intricate interplay between personal values, systemic limitations, and family vulnerability in EOL care.

## Case description

Mr. X was a male in his mid-50s with Glioblastoma treated with concurrent radiation and chemotherapy, followed by two cycles of adjuvant chemotherapy. His course was complicated by severe headaches associated with vasogenic edema and seizure episodes requiring multiple hospitalizations, including an intensive care unit (ICU) stay for airway protection and transfers to post-acute care facilities for rehabilitation. Mr. X received a brief course of second-line treatment upon disease progression, which was held because of wound complications and insurance coverage denial.

Supportive care (SC) was consulted 6 months after the initial diagnosis and followed the patient through his disease progression for symptom management, including pain, fatigue, emotional and financial distress, and caregiver support. When hospice was recommended by his oncologist due to progression of disease, SC engaged in multiple goals of care discussions to facilitate a safe transition.

During this hospitalization, the patient became more obtunded and started not responding to tactile or verbal stimuli. Mrs. X initially resisted psychological referrals and code status changes, opposing home hospice because of the potential emotional impact on her autistic adult son and her own physical limitations, including having to go to work daily. Despite reassurance that a Do Not Resuscitate (DNR) order would not reduce care quality, Mrs. X would not consider changing the patient’s code status until transfer to the Palliative and Supportive Care Unit (PSCU) or inpatient hospice, as the place of death was guaranteed. She shared with the medical teams that Mr. X’s wish was to die in the hospital.

After several days, Mrs. X accepted Supportive Care Psychology for caregiver support. During counseling, Mrs. X was able to share the overwhelming demands she faced: being the sole financial and health insurance provider, working full-time without any further sick leave, and caring for both her husband and their autistic adult son. With limited outside help, she remained relentless in advocating for her husband’s continued inpatient hospitalization to focus on comfort and safety.

Mrs. X acknowledged her husband’s poor prognosis but grew increasingly frustrated with barriers to honoring his wish of dying in the hospital rather than at home or in a skilled nursing facility. Her exhaustion, anticipatory grief, and fiercely independent nature contributed to defensiveness and persistent advocacy, which medical staff at times perceived as uncooperative. As administrative pressure for discharge planning mounted, she became more argumentative, filed complaints, and sought to “fire” providers she felt contradicted her stance. An initial meeting with the psychology team through our Ensuring Children Have Optimal Support (ECHOS) program, intended to support her son with autism, was misunderstood as a threat to her guardianship, further escalating her distress.

During a subsequent in-person visit, clarification and validation helped ease her defensiveness. A simple, empathic statement, “I can’t imagine how exhausted you must be doing all of this on your own,” shifted the dynamic, allowing her to speak more openly. She expressed fears about her inability to manage care at home, the potential impact on her autistic son if her husband died there, and the burden of feeling she was the only advocate for his hospital-based death. Building rapport revealed the depth of her stress and isolation, reframing her advocacy as an expression of love, grief, and profound strain.

The following day, Mrs. X agreed to change the patient’s code status to DNR and transition him to comfort care. By the time she made the decision, the patient had a profound clinical decline and became an appropriate candidate for the PSCU, requiring acute symptom management focused on comfort measures. The patient died one day after the initiation of the PSCU transfer. Mr. X’s length of hospital stay was 31 days.

## Discussion

The case reflects the intersection of caregiver burden, limited social support, and complex medical advocacy during a deeply emotional and sensitive EOL phase of illness. It also emphasizes that while physical care is a necessity, patients’ and families’ EOL needs are much broader and individualized. Psychosocial needs must also be assessed and incorporated into what then becomes a highly individualized EOL care plan (Steinhauser et al. 2000). Quality metrics such as “hospital death” as negative and undesirable outcomes may become barriers to personalized patient and family care (Tang and Bruera 2020).

Mrs. X’s decisions were shaped not only by her husband’s medical condition but also by trying to honor his wishes, overwhelming responsibilities, financial constraints, limited caregiving capacity, and the psychological well-being of her family, particularly that of her young adult son with autism. During the patient’s month-long hospitalization, there were numerous goals of care discussions, shifting from the hope for further treatment to changing code status and reviewing comfort care options.

The progression from initial resistance to acceptance of comfort measures and DNR status reflects a deeply conscientious process influenced by trust-building conversations, ethical consultation, and a desire to do right by her husband without compromising the rest of her family’s stability. Her decision-making revealed strong values around maintaining dignity and avoiding suffering, tempered by practical realities of limited options and distrust in home care capacity.

When asked about the preferred place of death, most cancer patients expressed a desire for a home death. However, these same patients had a dramatic change in choice when presented with different contexts, such as high symptom burden or the limited number of home visits they were likely to receive (Valentino et al. 2023). One hundred cancer patients receiving palliative care were asked to rank 35 priorities for end of life using the Go – wish – game (Delgado-Guay et al. 2016). Dying at home ranked 28th out of the 35, well below dimensions such as spirituality, physical comfort, connectedness, and not being a burden to their loved ones.

Currently, available hospices provide limited care (Tang and Bruera 2020), and patients and families should be made aware of these limitations before hospice referral and discharge. In this case, by remaining in the hospital until death, the patient was able to receive daily palliative care visits to ensure comfort, while his wife benefitted from psychosocial support and was able to maintain her dual role as mother and financial provider. This also underscores the shortcomings of EOL care outside the hospital, where the burden of daily care often falls heavily on family, particularly when skilled nursing facilities are financially inaccessible. More research is needed to define quality metrics that promote person-centered care and to identify gaps in the medical system that hinder achieving the highest quality of life at the EOL.
