# “He needs to die in the hospital”: A caregiver’s distress call – CORRIGENDUM

**DOI:** 10.1017/S1478951526102958

**Published:** 2026-07-10

**Authors:** Monica T. Agosta, Juleen Min, Imelda Pangemanan, Sujin Ann-Yi, Eduardo Bruera

**Keywords:** Case report; palliative care; end of life care; caregiver burden; hospital death; corrigendum

The Authors apologise for accepting the paper as is, without the additional names of secondary authors. The omitted author names are included, as follows, and they have now been added to the published article.

Monica T. Agosta^1^, PsyD; Juleen Min, MD^1^; Imelda Pangemanan, APRN; Sujin Ann-Yi, PhD; Eduardo Bruera, MD
